# The Effects of Hospital-Based School Lessons on Children’s Emotions, Distress and Pain

**DOI:** 10.5334/cie.118

**Published:** 2024-07-15

**Authors:** Enrica Ciucci, Lucrezia Tomberli, Elena Amore, Andrea Smorti, Francesca Maffei, Laura Vagnoli

**Affiliations:** 1 Dept. FORLILPSI, University of Florence, Italy; 2 University of Florence, Italy; 3 Meyer Children’s Hospital IRCCS, Florence, Italy; 4 Northwest Tuscany USL, Italy

**Keywords:** Hospital-based School, hospitalized children, emotions, pain, distress

## Abstract

Lessons conducted in hospitals ensure school continuity for hospitalized children unable to attend regular school. Hospital-based school (HS) provides a tailored experience that ensures normality for children through education. The objective of this study is to evaluate the effects of the proposed lessons in reducing negative emotions, distress, and pain in children, as well as fostering positive affects. The study was conducted with 32 hospitalized children, aged 8–12 years, in the Onco-Hematology and Pediatric Unit of Meyer Children’s Hospital IRCCS (Florence, Italy). Positive and negative emotions were measured using the *Positive and Negative Affect Scale for Children*; distress was measured using the *Physiological Hyperarousal for Children*; pain was measured using the *Visual Analogue Scale* for children. Variables were assessed before (T0) and after (T1) lessons, for three times; for each variable, collected data were averaged at both T0 and T1. Statistical analyses showed a significant increase in positive emotions in hospitalized children and a significant decrease in negative emotions, distress, and pain; nevertheless, only for pain the significant correlation between its scores before and after the HS lessons indicated that the detected change occurred for all participants in much the same way. These preliminary results suggest that HS lessons can promote hospitalized children’s well-being, at least as far as pain reduction is concerned.

Hospitalization can represent a stressful time for children who often experience suffering and discomfort caused by medical treatment (Coyne, 2006; Orenius et al., 2018; Wilson et al., 2010). Moreover, frequent and/or extended hospitalization can adversely affect academic and social functioning of children with significant medical issues, moving them away from their daily life at school and forcing prolonged periods at home (Lum et al., 2017; Prevatt et al., 2000; Rennick & Rashotte, 2009; Steinke et al., 2016; Vance & Eiser, 2002). Existing literature has shown that school is a source of well-being for children’s healthy and positive development, therefore, the Hospital-based School (HS) is offered to improve hospitalized children’s quality of life (Løhre, Lydersen, & Vatten, 2010; Tobia et al., 2019).

The positive association between poor health in childhood, adolescence, school absenteeism, academic underachievement, and socioemotional problems has been documented in a growing body of research (Allen et al., 2018; Allison & Attisha, 2019; Forrest et al., 2011; Lewallen et al., 2015). Frequent and/or extended hospitalization can be strongly associated with school dropouts, school difficulties such as less academic engagement and learning motivation (De Ridder et al., 2013; Emerson, et al., 2016; van Heesch et al., 2012), feeling of loneliness and lower levels of sense of school belonging. Further, hospitalization can compromise relationships with peers and teachers. It is also important to take into consideration that children in hospital are exposed to painful and numerous medical procedures with severe psychological distress, identified as aspects that impact psychological well-being (French et al., 2013; Friedrichsdorf & Goubert, 2021; Rokach, 2016; Steinke et al., 2016). Several studies have shown that after hospitalization chronically ill children can suffer of behavioral, emotional, and social problems, hyperactivity, and poor social behaviors (Brand, Wolfe & Samsel, 2017; Lum et al., 2019; Thompson et al., 2015; Yilmaz et al., 2014). HS offer children the opportunity to develop more social interaction (i.e., with children, teachers, educators, and peers) and it can reduce difficulties during school reintegration (Ratnapalan, 2009; Vance & Eiser, 2002; Tomberli & Ciucci, 2021). HS represent an opportunity for the child to feel less lonely, more connected with peers and friends, more “normal” and equal to peers (Capurso, di Castelbianco, Di Renzo, 2021; Sandeberg et al., 2008; Sullivan, Fulmer, & Zigmond, 2001; Tomberli & Ciucci, 2021).

HS can be found in many pediatric hospitals in the world with different organization and structure depending on the specific country; for example, HOPE (*Hospital Organisation of Pedagogues in Europe*)^1^ reports that HS’s service is offered in many countries in Europe (e.g., Belgium, Germany, Finland, New Zealand)^2^ and in the U.S.A (e.g., one of the biggest is the *Royal Children’s Hospital*).^3^ “In Italy, all pediatrics departments have a hospital school section with infant, primary, lower- and upper-secondary school teachers” (Benigno & Fante, 2020, p. 37). HS can be carried out in specific classrooms or at the student’s bedside; as reported from *Italian Ministry of Education University and Research*,^4^ most of the lessons take place one to one (pupil-teacher) or in a small group. Lessons in the hospital differ from regular ones only in the structure and timing in which they are carried out. Teachers working with hospitalized children report that hospital schooling and homeschooling help the student to achieve educational objectives, to feel “normal”, to imagine life in the future outside the hospital and to feel less lonely (Tomberli et al., 2023).

To date, studies have mainly addressed the role HS has on children’s education, but rarely the one it has on children’s quality of life (Caggiano et al., 2021; Dixon, 2014; Leger, 2014; Shaw & McCabe, 2008). Existing literature shows that attending traditional schools could be correlated with a better health-related quality of life for children under cancer treatment (Sandeberg, Johansson, Björk and Wettergren, 2008; Sodergren et al., 2017; Weibel et al., 2020), likewise, it is plausible to hypothesize that attending HS promotes positive health-related quality of life for sick children too.

Considering the known detrimental effects that hospitalization has on children with chronic medical conditions, besides their physical state, nationwide pediatric healthcare proposes to include comprehensive psychosocial care as a component of children’s medical treatment (Colbert, Edlynn, Mueller et al., 2020; Steinke et al., 2016). For example, more and more hospital settings are implementing psychosocial practices such as hospital clowning, animal assisted intervention (AAI) and musical activities that can have a complementary role in healthcare by easing the recovery of hospitalized children and validating their wellbeing (Bert et al., 2016; Lopes-Junior, Bomfim, Olson, Neves, Silveira, Nunes et al., 2020). Consequently, these practices can help reduce pain and anxiety (Atzori et al., 2018; Bradt, Dileo, Grocke, Magill, 2016; Braun et al., 2009; Dionigi, 2017; Felluga, Rabach, Minute, Montico, Giorgi, Lonciari, Taddio, Barbi, 2016; Vagnoli et al., 2005; Vagnoli et al., 2019) and also boost positive affects (Antonelli et al., 2019; Uglow, 2019), both during medical procedures and in caring for hospitalized children with chronic illness.

As far as we know, there is no existing research that investigates if attending HS lessons makes the child feel better and reduces pain and distress. To fill this gap, this study aims to examine if attending HS lessons promotes positive emotions by reducing negative emotions, distress, and perceived pain in children hospitalized in Oncology and Pediatric wards.

## Method

### Participants

A total of 32 Italian children (56.25% females) were recruited in the Onco-Hematology Unit (75%) and in the Pediatric Unit (25%) of Meyer Children’s Hospital IRCCS (Florence, Italy) (see Table 1). Participant recruitment criteria required:

**Table 1:** Sociodemographic Characteristics of the Participants.


SAMPLE CHARACTERISTICS	n	%	M	SD

Gender				

Male	14	43.75		

Female	18	56.25		

Onco-Hematology				

Male	9	28.12		

Female	15	46.88		

Pediatrics				

Male	5	15.63		

Female	3	9.37		

Age				

Male			9.86	1.03

Female			10.33	1.50

- Frequent visits to the hospital; Italian laws define the long stay as a hospitalization that lasts longer than 30 days. By law, the lessons in the HSs are activated even if the 30 days of hospitalization are not continuous due to the possibility that the student undertakes therapy on a daily basis, as opposed to being hospitalized for a period of time, and is discharged within a 24-hour period yet loosing many school days (Ministero dell’Istruzione, dell’Università e della Ricerca, 2019).
- An age range of between eight and twelve, since the questionnaires for evaluating children’s positive and negative affects and distress were validated for this age range.
- Being fluent Italian speakers.
- Not having cognitive and/or developmental impairments.

All children with the above-mentioned inclusion criteria took part in the study. There were no dropouts during the study.

### Instruments

#### Positive and Negative Affect Scale for Children (PANAS-C)

The Italian version (Ciucci, et al., 2017) of the *Positive and Negative Affect Scale* for Children (PANAS-C, Laurent et al., 1999) was used to evaluate Positive Affect (PA) for example, *happy, strong*, and *daring* and Negative Affect (NA) for example, *sad, frightened* and *lonely* in children.

The 11-item PA scale reflects the level of pleasant engagement and high activation, which means that it measures how much a person feels excited, enthusiastic, concentrated, active, and determined. The 13-item NA scale reflects the level of unpleasant engagement and subjective distress, that includes a broad range of aversive affects including distress, fear, nervousness, guilt, and shame. Participants rated how much they were experiencing the affects on a 5-point scale (from 1 = very slightly or not at all to 5 = extremely), which referred to the here and now following a request to report the intensity of their current affects. For each participant, the mean scores for the 11-item PA scale and 13-item NA scale were calculated, assessing the level of the child state affects in terms of pleasantness and unpleasantness. On PANAS-C_PA subscale, higher scores reflect higher levels of intensity of pleasant engagement and high activation; on PANAS-C_NA subscale, higher scores reflect higher levels of intensity of unpleasant engagement and subjective distress. The average values of the scores of PANAS-PA and PANAS-NA collected before and after three HS lessons were calculated.

#### Psychological Hyperarousal Scale for Children (PH-C)

The Italian version (Ciucci et al., 2017) of the *Psychological Hyperarousal Scale for Children* (PH-C, Laurent, Catanzaro, & Joiner, 2004) was used in this study to assess physiological hyperarousal (the bodily manifestation of autonomic arousal) as an increased level of bodily distress. The Italian version of PH-C is a 14-item self-report measure; children were asked to rate the extent of the physiological symptoms they were experiencing “right now” on a 5-point Likert scale (from slightly or not at all to extremely) (e.g., *Feeling of choking, Feeling like throwing up, Pain in the chest*). The mean score for the 14-item PH-C scale was then calculated for each participant. On PH-C scale, higher scores reflect higher levels of intensity of bodily distress. The average value of the scores of PH-C collected before and after three HS lessons were calculated.

#### Visual Analogue Scale (VAS) for children

The *Visual Analogue Scale (VAS)* for children, by Scott, Ansell, & Huskisson (1977), is a self-rating measure of pain used with children older than 6 years and it investigates the level of the pain they are feeling “right now”. It is shaped like a thermometer ranging from 0 (*no pain at all*) to 10 (*the worst pain imaginable*). The raw score was reported for each participant. On VAS scale, higher scores reflect higher levels of intensity of pain. The average value of the scores of VAS, both collected before and after three HS lessons, were calculated.

### Procedure

Hospital Committee approval was obtained in March 2017, data were collected until February 2020 (before the Covid-19 pandemic), and lessons were conducted by teachers while the researchers administered the instruments without making any adjustments.

Therefore, the questionnaires were administered before the lesson and at the end of it.

To enroll participants in the study, two Pediatric Psychology of Meyer Children’s Hospital IRCCS identified children who met the study’s inclusion criteria; then, one hospital psychologist and a research psychologist of the study required parents to sign the informed consent so the children could participate in the study.

As for HS experiences at Meyer’s hospital, similarly to many HS experiences in the world (Ratnapalan et al., 2009; Steinke et al., 2016), HS teachers usually provided one-on-one lessons either in a hospital classroom or at the child’s bedside and adapt each lesson to each student’s needs. Specifically, HS teacher taught at the bedside of the child 92% of the times, with the remaining time facilitating lesson one-on- one in the HS classroom. Lessons lasted 20–60 minutes depending on child’s health issues and medical visits and therapies. All the lessons were structured as follows: the first part was dedicated to teaching a new content whereas, the second part to verifying what was understood through oral questions. The teachers were free to use all the materials they wanted during the lessons such as books, notebooks, as well as tablets. However, all the teachers used books and notebooks regardless of the subject. The school subjects proposed during classes were math, geometry, history, Italian, English, geography, and science. During lessons, parents were asked to go out of the room to allow their child to freely have lessons with the teacher without interference.

Before starting each lesson (T0), the children were asked to complete three questionnaires to evaluate positive and negative affects (PANAS-C), physiological hyperarousal (PH-C) and pain (VAS). Then, they were asked to fill out the questionnaires again after the lesson (T1). While children were required to complete them individually, there were no time limits to complete the questionnaires. Thus, only the researchers and the child were present in the room during the survey.

This procedure was repeated on three separate days by the same research psychologist; in order to partially take of control variable conditions of the lessons, it was decided to measure the average of the scores on the study variables before and after the three different lessons. The confidentiality of answers on the questionnaires and the associations among the three different administrations were guaranteed by replacing the name of each participant with the same alphanumeric code.

Data collection took a long time because when lessons were interrupted by medical visits or checkups, data were not recorded. This happened an average of two times for each child. Therefore, to obtain five surveys it was necessary to participate in at least five lessons for each child. 75% of the interrupted lessons were suspended because of the child’s medical therapy and 5% because of in-depth medical examinations. The interrupted lessons were excluded from the study for two reasons; medically in some cases it was not possible to ask children to fill in the questionnaires after medical visits or treatments because these had tired them a lot and because the medical visit would have confused the interpretation of the research data; measuring the emotional experience of the child after the interruption could mean measuring the effects of what the child had experienced during medical examination rather than the effects of the lesson attended. Ultimately, only 32 hospitalized children were able to attend the three lessons, without any interruption.

### Data Analysis

Data analyses were carried out on the final sample of the 32 hospitalized children. Descriptive statistics (along with values of skewness and kurtosis) of the average study variables before (T0) and after HS lessons (T1) were calculated, demonstrating a normal distribution of scores.

To evaluate the influence of the HS lessons on children’s affects, distress and pain, paired sample *t* tests were used. Specifically, the paired samples *t-*test compared the means of measurements of PANAS-C PA, PANAS-C NA, PH-C and VAS taken from the same individual at two different times (e.g., before HS lessons, T0 score and after HS lessons, T1 score); when the *p* (probability) value associated to the *t* is significant (≤.05), we can conclude that the means are significantly different.

For each pair of variables entered in *t* test, correlation coefficient was also calculated (i.e., bivariate Pearson): in the case of a significant difference at the *t* Test and a significant correlation between the variable at T0 and T1, the result indicates that the significant detected change occurs for all participants in much the same way.

All the analyses were conducted using SPSS 25 for Windows (SPSS Inc, Chicago, IL).

## Results

In the Table 2 paired samples *t*-test results on the Study Variables are reported. As for positive affects (PANAS-C PA), we found that the mean score at T1 is significantly higher than the mean score at T0; scores at two assessments were not correlated (*r* = –.12, *p* = .52), suggesting that an increase in the level of pleasant engagement and high activation is not consistent across all children. As for negative affects (PANAS-C NA), we found that the mean score at T1 is significantly lower than the mean score at T0; the scores at two assessments were not correlated (*r* = .28, *p* = .13), suggesting that a decrease in the level of unpleasant engagement and subjective distress is not consistent across all children. As for physiological distress (PH-C), we found that the mean score at T1 is significantly lower than the mean score at T0; the scores at the two assessments were not correlated (*r* = .16, *p* = .39), suggesting that a decrease in the level of bodily distress is not consistent across all children. As for pain intensity (VAS), the mean score at T1 is significantly lower than the mean score at T0; the scores at the two assessments were strongly and positively correlated (*r* = .76, *p* ≤ .001), suggesting that a decrease in the level of pain condition is strongly consistent across all children.

**Table 2:** Paired samples t-test results on the Study Variables.


	T0		T1		t-TEST	p-VALUE
	
	M	SD	M	SD		

**Panas-C_PA**	2.25	.09	3.37	.11	–42.60	**<.001**

**Panas-C_NA**	2.46	.15	1.15	.04	51.58	**<.001**

**PH-C**	2.11	.10	1.18	.05	50.45	**<.001**

**VAS**	2.26	.89	.85	.62	13.74	**<.001**


*Notes:* PANAS-C_PA: Positive Affect Subscale for Children; PANAS-C_NA: Negative Affect Subscale for Children; PH-C: Psychological Hyperarousal Scale for Children; VAS: Visual Analogue Scale; T0: before the HS’ lessons; T1: after the HS’ lessons. In bold p-value < 0.05.

## Discussion

The present study aimed to examine if attendance to HS lessons contributes to promote positive emotions, reducing negative emotions, distress, and perceived pain in children in oncology or pediatric wards. Results revealed that attending HS classes significantly increases positive emotions and reduces negative emotions, distress and pain in 8–12-year-old hospitalized children in oncology or pediatric wards. These preliminary results suggest that hospital school lessons can promote hospitalized children’s well-being in so far as pain reduction is concerned. Nevertheless, results about correlations between the two assessments before and after HS lessons indicated that change in positive and negative emotions as well as in distress did not occur for all participants in much the same way, while the change that affected pain occurred in the same way for most of the children.

Growing evidence about practices performed in hospital settings according to a comprehensive psychosocial care to hospitalized children (Steinke et al., 2016) such as hospital clowning, Animal Assisted Intervention, and musical activities revealed the efficacy of these practices in reducing children’s pain and anxiety (Braun et al., 2009; Vagnoli et al., 2015) whilst empowering children’s wellbeing (Antonelli et al., 2019). As far as we know, this is the first study aimed to investigate the efficacy of HS lessons attendance in changing affects (emotions), distress and pain in hospitalized children. The present study provides preliminary data which suggests that HS lessons have an impact on hospitalized children’s well-being in the same way regular school attendance does for healthy children (e.g Sandeberg et al., 2008). Our results showed statistically significant effects of HS classes for increasing positive emotions, decreasing negative emotions, distress, and pain, with variable effects in 8–12-year-old hospitalized children within oncology or pediatric wards. These results appeared promising since the literature on regular schooling has shown that experiencing positive school-related emotions reduces dropout rates and psychological difficulties over the course of children’s development (Boekaerts & Pekrun, 2015; Valiente et al., 2012).

Unlike clown therapy, music therapy, and animal assisted activities, HS lessons require more continuity over time and a greater focus on the child in didactic tasks rather than being an amusement or distraction. Furthermore, according to the literature on regular schools, we can hypothesize that an important role of the children’s well-being in school may be represented by the relationship with teachers (Hallinan, 2008; Kennedy & Kennedy, 2004; Løhre, Lydersen, & Vatten, 2010; Raikes, 1993; Riley, 2010; Verschueren & Koomen, 2012). In fact, the child who attends HS lessons could develop a rather strong relationship with the hospital teacher due to the frequent one-on-one teaching condition (Capurso & Dennis, 2017; Tomberli & Ciucci, 2021; Tomberli et al., 2023). Nevertheless, research is needed to clarify these aspects. Furthermore, we wonder if the effectiveness of HS lessons cannot also in part depend on whether attending actively and continuously classes at HSs allow the young person to take part in something other than the illness and to feel part of something else. With respect to the topic of Hospital-based School, the review of Tomberli and Ciucci (2021) highlighted how it is important for hospitalized students to feel connected to the school so that certain educational and relational objectives are achieved. In the future, we could investigate whether this sense of school belonging also takes place while attending HS according to Bronfenbrenner’s Theory of Ecological Systems (2005) which states that carrying out a regular activity for a prolonged time affects individuals’ emotional and relational development.

Further studies could be focused on the differences occurring within HSs and other activities often opted for within the hospital environment such as, watching TV or playing video games which students engage in over a prolonged period of time; consequently, this aspect does not seem to have been explored in the literature to date.

### Implications for school context

Data collected show the importance of considering scholastic experiences for hospitalized children. Most of the studies regarding pediatric hospitalization have focused on psychosocial practices (e.g., hospital clowning, AAI, and musical activities) and useful tools to promote greater well-being in hospitalized patients. The findings of the current study propose that HSs may serve as a significant contributor to enhancing the quality of life for children experiencing intermittent absences from conventional schooling. Despite the small sample size, the results indicate that HS lessons have the potential to mitigate distress and discomfort while fostering positive emotional experiences.

Experiencing less psychological discomfort during hospitalization reduces school dropout and limits psychological consequences such as anxiety, depression, learning or behavioral disorders. Therefore, investing economically in school well-being of hospitalized children also means investing in the well-being of children, their family members and society as well.

## Conclusion

The study expands the current literature on HS considering not only its impact on academic and social functioning of hospitalized children but also on their emotional functioning. The study investigated a specific hospital setting and therefore, although the results are promising, it is conceivable that there may be differences between one hospital school and another. Moreover, the lessons were conducted in a predominantly one to one ratio, which could lead to hypothesize that the hospital teacher-pupil relationship may constitute a moderation variable, with respect to the study variables taken in account, in line with what happens in regular schools along with what is highlighted in the literature on teacher attachment.

The results carried out in this paper reinforce the idea that hospital schools are not only useful from an emotional and educational point of view, but also from a clinical one with the aim of reducing pain. This is an important finding promoting the idea that schools have an impact not only on students’ mental health, but also on clinical outcomes. These aspects deserve to be explored further in the future.

Also, it is important to signal that children who agreed to take part in this study were the ones who felt better, while those who felt worse turn down the invitation or they were not listed in first place as possible participants by the Pediatric Psychology of the Hospital; yet, it is worth noticing that recruiting children in oncology or pediatric wards can be problematic.

The study is not free of limitations. Given the current small sample size, these data represent preliminary efforts to evaluate the effectiveness of HS lessons on hospitalized children’s emotional functioning. As the number of participants increases in subsequent studies, additional analyses should be performed to investigate in depth the influence of the unique role the relationship with the hospital’s teacher has on the emotional experiences of the hospitalized children; also, it would be interesting to compare HS with other psychosocial practices such as hospital clowning, animal assisted intervention and musical activities – or to other regular and continuous activities – such as watching TV or playing video games – in order to measure the unique role that attending HS lessons can have on children’s emotional functioning during hospitalization. Moreover, further studies could jointly consider the impact of HS lessons on educational, social and emotional functioning of hospitalized children.

The present study cannot use a control sample because the one-to-one classes in HSs and the reduced educational program provided by teachers to hospitalized children are not comparable with usual organization in regular schools. In hospital settings, it is very difficult to keep several variables under control such as those related to medical treatment participants received at the time of the study that may have also contributed to increased or improved affects it is very time-consuming recruit children with similar medical issues. Moreover, it was necessary to consider that the first lesson for a child could be the 10th lesson for another child and so on. Therefore, assessments repeated three times on each child allowed measuring the average variation across affects, distress and pain.

Furthermore, the time interval between lessons varied because of logistic issues. That is, in the oncological and pediatric wards each child gets different medical treatments and comes to hospital at varying intervals (there are children that only experience day hospitals or recoveries and others who have to stay in hospital for long periods). Hence, it is not possible to predict when children are coming back for treatment or when they do, that they will be willing to cooperate. For these reasons each child was tested three times regardless of the data collecting time. In the future, it will be interesting to continue an in depth evaluation of the HS role in order to ensure comprehensive support for the adjustment of hospitalized children.

## Footnotes

https://www.hospitalteachers.eu.

https://www.hospitalteachers.eu/who/links/schools.

https://www.rch.org.au/education/.

https://scuolainospedale.miur.gov.it.
